# Step Counts of Middle-Aged and Elderly Adults for 10 Months Before and After the Release of Pokémon GO in Yokohama, Japan

**DOI:** 10.2196/10724

**Published:** 2019-02-05

**Authors:** Kimihiro Hino, Yasushi Asami, Jung Su Lee

**Affiliations:** 1 Department of Urban Engineering Graduate School of Engineering The University of Tokyo Bunkyo-ku, Tokyo Japan; 2 Department of Public Health Graduate School of Medicine The University of Tokyo Bunkyo-ku, Tokyo Japan

**Keywords:** augmented reality, location-based games, mobile phone, pedometer, physical activity, Pokémon GO, smartphone

## Abstract

**Background:**

Smartphones have been integrated into our society and are expected to serve as tools to improve health outcomes. In the summer of 2016, Pokémon GO, a location-based augmented reality game for smartphones was released; it attracted attention from the perspective of health, especially with its potential to increase physical activity (PA). A few studies have compared objectively measured step counts before and after the release of the game; however, they were conducted over a short study period and evaluated only young people.

**Objective:**

The objective of this study was to confirm whether there was a difference in step counts between middle-aged and elderly players and nonplayers before and after the release of Pokémon GO.

**Methods:**

A total of 46 players and 184 nonplayers aged ≥40 years were matched for sex, age group, and PA level; they were respondents to a questionnaire randomly sent to citizens who were given free pedometers by Yokohama city. Their play status was identified through the questionnaire. To investigate the change in step counts before and after the release of Pokémon GO according to play status, a 2-way repeated-measures analysis of variance was performed. Step counts 1 month before the release of the game were compared with those 8 months after the release. In addition, subgroup analyses according to sex, age group, PA level, and subjective health status were performed.

**Results:**

The mean ages of players and nonplayers were 56.5 (SD 9.9) years and 57.3 (SD 9.6) years, respectively, and the mean baseline step counts of players and nonplayers were 7641.8 (SD 2754.5) and 7903.3 (SD 2674.7), respectively. There was no significant difference in the age and baseline step counts according to a t test (2-tailed). In the analysis of all samples, the interaction between play status and time effect was significant for 3 of 8 months after release. In the subgroup analyses, the interaction was significant for 3 months in men, 7 months in the 55-64-year-old group, 2 months in workers, 4 months in the active group in PA level, and 2 months in participants with subjectively good health. The interaction was significant for only 1 month, at most, in other subgroups.

**Conclusions:**

The present study confirmed a difference in step counts between players and nonplayers before and after the release of Pokémon GO. According to our analysis, step counts were higher until 7 months after the release. The player group maintained their step counts in winter, despite the decrease in step counts of nonplayers. In subgroup analyses, players were more likely to be men, aged <55 years, workers, active, and subjectively in good health.

## Introduction

### Background

Smartphones have become integrated into our society and can serve as tools to improve health outcomes [1], especially in increasing physical activity (PA), which, in turn, is associated with physical and mental health [2]. However, until a few years ago, most apps incorporated few behavioral change techniques, such as feedback on behavior [3,4], and only a few studies have assessed the effects on PA of interventions using smartphone technology [5-7].

Pokémon GO is a location-based augmented reality game for smartphones released in the summer of 2016. According to the Guinness World Records, it was the “most downloaded mobile game in its first month” [8] and was the number 1 free app in 36 countries [9]. Although the combination of location-based service and augmented reality is not novel, Pokémon GO attracted attention because of the perfect incorporation of technology into the story within the game [10]. Players are encouraged to walk outside to catch Pokémon characters, hatch their eggs, and acquire game items by visiting Pokéstops and Gyms.

The game has attracted attention from the perspective of health [11-16] as a “game changer” for the physical inactivity crisis [17]. Pokémon GO players collectively walked 4.6 billion kilometers in only 8 weeks, surpassing 500 million downloads worldwide [18]. Twitter postings demonstrated that Pokémon GO players walked >30 minutes a day [19], and the game even changed peoples’ hot-spots [20] and the general mobility patterns [21] in cities.

### Previous Studies

For Pokémon GO players, health or PA is an important and unique motivation to play, which is not usually the case in other Web-based gaming [22-24]. In addition, positive outcomes have been observed in not only PA seekers but also players with other motivations for playing [25].

From the viewpoint of psychological health, Pokémon GO moderated psychological distress [26] and social anxiety [27], as well as increased place attachment [28]. These psychological factors relate to behavioral consequences; enjoyment of Pokémon GO remarkably resulted in perceived increases in outdoor PA [29].

Some studies used the International Physical Activity Questionnaire to report an increase in PA in players after playing Pokémon GO [22,30,31]. Several other studies used different questionnaires and reported an increase in PA frequency and duration [32], increase in PA and decrease in sedentary behaviors [33], and increase in time spent walking dogs [34]. However, self-serving bias cannot be avoided when using self-reported measurements [35-37].

Some studies have compared objectively measured step counts using a pedometer or an accelerometer, which can explain most of the variation in PA [38] before and after playing Pokémon GO. One study compared the number of daily steps taken by players and nonplayers for 4 weeks before and 6 weeks after installation of Pokémon GO. That study concluded that step counts moderately increased after installation of the game, but this result was no longer observed 6 weeks after installation [39]. Another study compared players’ step counts 3 weeks before and 3 weeks after the Pokémon GO release date and observed the largest increases in participants who spent more time playing, who were overweight or obese, or who had a low baseline PA level [40]. However, both of the abovementioned studies measured the number of step counts using the iPhone Health app, which has considerable difference from the accelerometer under free-living conditions [41]. Another study used Microsoft Band, which includes a 3-axis accelerometer and gyrometer, and observed that players identified through Microsoft’s search engine queries had notably increased PA over an observation period of approximately 4 weeks [42].

### Study Aim

This study aimed to confirm whether there was a difference in step counts between middle-aged and elderly players and nonplayers before and after the release of Pokémon GO in Japan. The long-term impact of the game on PA should be examined; however, previous studies have evaluated its impact for only several weeks [42,43]. The uniqueness of this study lies in its long survey period (10 months), use of a pedometer, and focus on middle-aged to elderly people, while most previous studies focused on younger people [30,31,33,44]. As PA patterns change by age [45], the impact of the game for middle-aged people, who tend to be more occupied with social responsibilities, and for elderly people after retirement is expected to be different than the impact on younger people. Pokémon GO was released in Japan on July 22, 2016.

## Methods

### Yokohama Walking Point Program

Yokohama city is the second largest city in Japan, with a population of approximately 3.7 million and a humid subtropical climate with 4 distinct seasons. The city launched the Yokohama Walking Point Program (YWPP) in November 2014 to encourage citizens to improve their health and enjoy a healthy life expectancy, as the population ages and the disease structure changes in Japan.

A free pedometer (Omron HJ-326F, Japan) was given to citizens aged ≥18 years who volunteered to participate in the program. Participants were awarded points by scanning their pedometers via special readers installed at approximately 1000 stores and other facilities in the city. Accumulation of a certain number of points made participants eligible to win prizes. The scanned data were sent to a data server through the internet, and participants could monitor step counts and rank among all participants using a computer or a smartphone [46].

### Pedometer Data

Pedometer data, which were collected from 231,606 YWPP participants who registered before March 31, 2017, were used in this study. The daily mean step count for each month was calculated for each participant using the monthly step count and the number of days recorded for that month. Data on sex and date of birth were obtained from registered information, and age as of May 31, 2016, was considered.

### Questionnaire

In May 2017, Yokohama city conducted a questionnaire survey among 2580 participants, who were randomly selected among 99,462 individuals who sent their recorded data of >80% days after registration; among the selected participants, 2055 replied to the survey (response rate 2055/2580, 79.65%). The questionnaire data and the pedometer data were combined in the analysis.

The questionnaire mainly asked about the change in participants’ walking habit and health attitude as well as their use of “smartphone game apps in which players catch characters and occupy bases using location services.” Participants who selected the first of 4 options ranging from “frequently” to “not at all” in a question on the frequency of use of such smartphone game apps were regarded as “players” and others as “nonplayers” in this study. Regarding occupation, participants were classified into workers (including part-time employees) and nonworkers. While health status was graded according to 4 points ranging from good to poor, it was also classified into “good” and “others” because the latter two scales (rather poor or poor) were not often selected.

### Study Participants

Among the 2055 participants who replied to the questionnaire, those who had records of limited days or had abnormal records were excluded. First, 1079 participants with pedometer data for <10 days per month in any month from June 2016 to March 2017, except July 2016 (which was when Pokémon GO was released in Japan), were excluded. In addition, 62 participants whose step counts in any of those months were out of the range of the mean (SD 2) in all samples were excluded as outliers.

Among the remaining 914 participants, 46 (5.0%) were game players. Among nonplayers, 184 participants, which was equivalent to 4 times the number of players, were randomly selected so that the ratio of players to nonplayers was 1:4 after stratification by sex, age group (<55, 55-64, and ≥65 years), and PA level. We considered the age of 65 years as the threshold for elderly persons in Japan, with the employment rate decreasing the most at this age. Participants aged <65 years were divided into 2 groups so that the numbers of participants in the 2 groups were similar. The participants’ PA level was based on whether their baseline step counts were higher than the target in the Japanese national health promotion plan (men aged <65 years, 9000; women aged <65 years, 8500; men aged ≥65 years, 7000; women aged ≥65 years, 6000) [47]. Finally, a total of 230 participants were included in the analysis.

### Statistical Analysis

To investigate the change in step counts before and after the release of Pokémon GO according to play statuses, a 2-way repeated-measures analysis of variance was performed. Step counts in June 2016 (baseline) were compared with those for each month from August 2016 to March 2017. After analysis of all samples, subgroup analyses according to sex, age group, PA level, and subjective health status were performed. The effect of Pokémon GO could be determined based on the interaction between the play status and the time effect. The significance level was set at *P*<.05. Shapiro-Wilk test was used to assess the normality of the distribution of samples’ step counts in each group in each month. Levene test was used to assess the homogeneity of variances between groups in each month. All statistical analyses were conducted using IBM SPSS Statistics 23 (IBM Corp).

## Results

### Sample Statistics

Participants’ characteristics are presented in Table 1. The youngest participant was 40 years old because YWPP recruited citizens aged 18-39 years since June 2016. The mean ages of players and nonplayers were 56.5 (SD 9.9) years and 57.3 (SD 9.6) years, respectively. The mean baseline step counts of players and nonplayers were 7641.8 (SD 2754.5) and 7903.3 (SD 2674.7), respectively. No significant difference was observed between players and nonpalyers regarding age and baseline step counts using a *t* test (2-tailed; *P*=.56 and.61, respectively). Although the percentage of players among nonworkers was lower than that among workers, the difference was not significant according to chi-square test (*P*=.22). Regarding subjective health status, the proportion of participants in good health was almost the same in players and nonplayers.

### Analysis of All Samples

**Figure 1:**
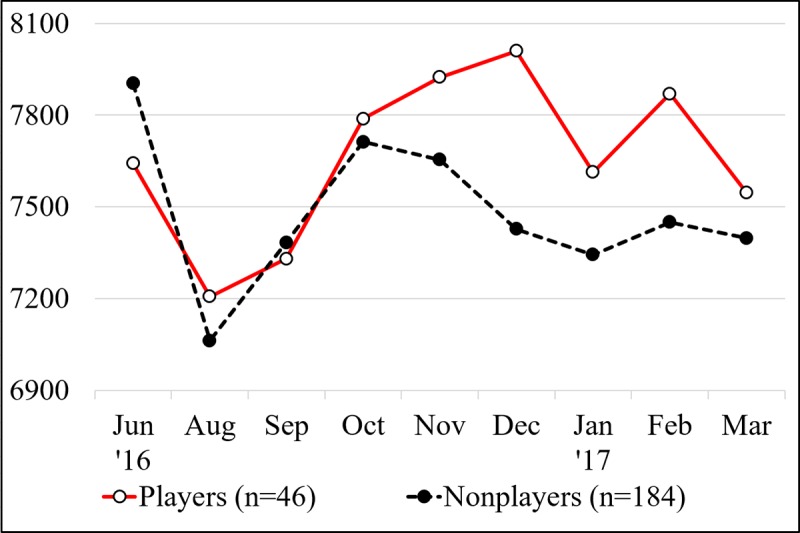
Mean daily step counts at baseline (June 2016) and follow-up (August 2016 to March 2017).

The results of the analyses of all samples are presented in Table 2. Although nonplayers walked more than players by 261 steps per day in June 2016 (baseline), the difference in step counts between the 2 groups got smaller between August and October, and players walked more than nonplayers later on. Time effect was significant in August, September, January, and March, when the step counts of participants decreased compared with that at baseline. The interaction between the play status and time effect was significant in November, December, and February. Although a decrease in step counts was observed in the nonplayer group in winter, the player group maintained their step counts (Figure 1). The largest difference in step counts between players and nonplayers was 583 steps per day in December.

**Table 1:** Characteristics of the study participants.

Characteristic		Pokémon GO Players, n (%)	Nonplayers, n (%)	Total, n
**Sex**				
	Male	23 (20.0)	92 (80.0)	115
	Female	23 (20.0)	92 (80.0)	115
**Age in years**				
	<55	20 (20.0)	80 (80.0)	100
	55-64	16 (20.0)	64 (80.0)	80
	≥65	10 (20.0)	40 (80.0)	50
**Occupation**				
	Worker^a^	33 (22.4)	114 (77.6)	147
	Nonworker	13 (15.7)	70 (84.3)	83
**Physical activity level^b^**				
	Active	17 (20.0)	68 (80.0)	85
	Nonactive	29 (20.0)	116 (80.0)	145
**Health status**				
	Good	17 (20.7)	65 (79.3)	82
	Others^c^	29 (19.6)	119 (80.4)	148
Total		46 (20.0)	184 (80.0)	230

^a^Worker included part-time employees.

^b^Participants’ physical activity level was based on whether their baseline step count was higher than the target in the Japanese national health promotion plan (men aged <65 years, 9000; women aged <65 years, 8500; men aged ≥65 years, 7000; women aged ≥65 years, 6000).

^c^Others health status included rather good, rather poor, and poor.

**Table 2:** Results of a 2-way repeated-measures analysis of variance investigating changes in step counts before and after the release of Pokémon GO according to play statuses (N=230).

Time point^a^		Players (n=46), mean (SD)	Nonplayers (n=184), mean (SD)	Time effect		Interaction term (time×player)	
				*F* test (*df_1_*, *df_2_*)	*P* value	*F* test (*df_1_*, *df_2_*)	*P* value
**Baseline**							
	June ’16	7642 (2754)	7903 (2675)	N/A^b^	N/A	N/A	N/A
**Follow-up**							
	August ’16	7207 (2888)	7063^c^ (2565)	23.20 (1, 228)	<.001	2.35 (1, 228)	.13
	September ’16	7331 (2546)	7383 (2479)	15.81 (1, 228)	<.001	1.00 (1, 228)	.32
	October ’16	7789 (2754)	7712 (2493)	0.04 (1, 228)	.84	2.46 (1, 228)	.12
	November ’16	7924 (3012)	7654 (2500)	0.02 (1, 228)	.90	4.12 (1, 228)	.04^d^
	December ’16	8010 (2946)	7427 (2434)	0.19 (1, 228)	.67	11.36 (1, 228)	.001^d^
	January ’17	7613 (3000)	7343 (2512)	4.16 (1, 228)	.04^d^	3.39 (1, 228)	.07
	February ’17	7870 (3086)	7450 (2497)	0.71 (1, 228)	.40	6.55 (1, 228)	.01^d^
	March ’17	7547 (2857)	7398 (2493)	5.39 (1, 228)	.02^d^	2.52 (1, 228)	.11

^a^No violation of homogeneity of variance assumption between groups in any month occurred.

^b^Not applicable.

^c^Significantly deviated from normality (Shapiro-Wilk test: *P*<.05).

^d^*P*<.05.

### Subgroup Analysis

The results of the subgroup analyses are summarized in Table 3 (the complete results are presented in Multimedia Appendix 1). The interaction was significant for 3 months in men, but only 1 month in women. Regarding age, the interaction was significant for 7 of 8 months in the 55-64-year-old group but no month in the other two groups. In the 55-64-year-old group, the difference between players and nonplayers amounted to 1891 steps per day in December, whereas the difference was 320 steps per day at baseline. Regarding occupation, the interaction was significant for 2 months in workers and 1 month in nonworkers. Regarding PA level, the interaction was significant for 4 months in the active group, but only 1 month in the nonactive group. Finally, regarding subjective health status, the interaction was significant for 2 months in participants with good health and 1 month in others.

**Table 3:** *P* value of interaction by subgroup analyses according to sex, age group, physical activity level, and subjective health status.

Time point	Sex		Age group (years)			Occupation			Physical activity level			Health status		
	Male (n=115)	Female (n=115)	<55 (n=100)	55-64 (n=80)	≥65 (n=50)	Worker (n=147)		Nonworker (n=83)	Active (n=85)		Nonactive (n=145)	Good (n=82)		Others (n=148)
August ’16	.28	.29	.35	.03^a^	.24	.08		.96	.02^a^		.80	.61		.13
September ’16	.58	.37	.43	.31	.71	.24		.87	.11		.96	.64		.35
October ’16	.36	.18	.60	.007^a^	.73	.19		.27	.02^a^		.81	.22		.31
November ’16	.03^a^	.64	.77	.005^a^	.77	.02^a^		.86	.11		.17	.02^a^		.54
December ’16	.02^a^	.01^a^	.31	.001^a^	.23	.02^a^		.01^a^	.01^a^		.02^a^	.01^a^		.03^a^
January ’17	.06	.67	.61	.02^a^	.94	.08		.39	.30		.13	.10		.31
February ’17	.006^a^	.48	.71	.008^a^	.24	.07		.08	.07		.07	.06		.08
March ’17	.09	.70	.93	.02^a^	.99	.12		.66	.02^a^		.84	.36		.20

^a^*P*<.05.

## Discussion

### Principal Findings

This study confirmed the difference in step counts between players and nonplayers before and after the release of Pokémon GO. On analyzing all samples, we observed that step counts were significantly high even in February 2017, 7 months after the release of the game, which was not observed in previous short-term studies. Players did not decrease their step counts even in cold months, unlike nonplayers and other YWPP participants [46].

In subgroup analyses, step counts of the 55-64-year-old group were significantly high until the last surveyed month. This finding, along with the result that significantly higher step counts were noted in men and workers than in women and nonworkers, indicates that older, middle-aged male workers may take long, indirect routes to visit Pokéstops and Gyms for catching rare Pokémon on their way to the work place, visiting other places, and while going home. This result is in accordance with a previous finding, according to which people did not change their daily routines but slightly modified them to play the game [21]. Although the higher step counts of players aged ≥65 years was not significant in any month in this study, some senior citizen’s clubs in Japan have promoted Pokémon GO to increase outdoor activities, as older people continue to play the game despite its diminishing popularity among younger players [48]. Such trials are recommended along with the development of additional location-based augmented reality games following Pokémon GO [49] for young people who lose interest quickly.

### Comparison With Previous Studies

Previous studies have observed short-term effects on the PA of young people [30,31,33,44]. For example, a US study conducted in survey participants aged 18-35 years reported that the difference in daily step counts between players and nonplayers was approximately 1000 in the first week after installation of Pokémon GO [39]. The difference in this study was smaller (583 steps in December, at most), which may be because the players were older and did not play the game as intensely as younger players or because the analysis was conducted for each month and a short-term difference may have been overlooked. We observed significantly higher step counts in more months in the active group than in the nonactive group, unlike a previous study in which it was reported that players with a lower baseline PA level had increased PA [40,42]. In addition, it may be due to the difference in sample characteristics and length of the study period.

### Limitations

Our study had some limitations. First, all characteristics of the study sample could not be simultaneously considered because of the limited number of participants who played the game. If there were more participants, we could have specified in more detail those who increased their step counts and continued to play the game. Their motivations for taking part in the YWPP, such as family structure and living area, should be considered as well. Second, the distribution of step counts in some subgroups did not meet the assumptions of normality and homogeneity of variance. The results of these analyses might not be as valid as those of others, although the *F* test is considered to be robust for nonnormal data [50]. Third, it is unclear whether the results of this study depend on the characteristics of the country, city, or participants. Further studies under different conditions are required to further examine the results. Fourth, there was a lack of variables regarding when, where, and why the participants played Pokémon GO, besides their intensity for the game, such as playing time or experience points in the game [40]. Finally, the effect on physical and psychological health, other than step counts, was not confirmed. Its negative impact, especially accidents [51-53] and injury [54,55], should be considered as well. However, our study demonstrated the potential of location-based augmented reality apps in promoting PA for a longer period than expected in the literature, at least for particular groups.

### Conclusions

This study confirmed the difference in step counts between players and nonplayers before and after the release of Pokémon GO. According to our analysis, step counts were higher until 7 months after the release. The player group maintained their step counts in winter, despite the decrease in step counts of nonplayers. In subgroup analyses, players were more likely to be men, aged <55 years, workers, active, and subjectively in good health.

## Appendix

Multimedia Appendix 1 The full results of subgroup analysis by sex, age group, physical activity level, and subjective health status.

## Abbreviations

PA: physical activity
YWPP: Yokohama Walking Point Program
