# Supplementary material for: Step Counts of Middle-Aged and Elderly Adults for 10 Months Before and After the Release of Pokémon GO in Yokohama, Japan
Source: J Med Internet Res. 2019 Feb 5;21(2):e10724. doi: 10.2196/10724 (PMC6379816; doi:10.2196/10724)
Supplement: Multimedia Appendix 1 [file jmir_v21i2e10724_app1.pdf]

Multimedia Appendix 1

The full results of subgroup analysis by sex, age group, PA level and subjective health status.

Table A1. Results of a 2-way repeated-measures analysis of variance (Male; N=115)

Time point		Players (n=23)		Nonplayers (n=92)		Time effect		Interaction term (time×player)	
		Mean	SD	Mean	SD	*F* test (*df*)	*P* value	*F* test (*df*)	*P* value
Baseline	16 Jun	8202	3140	8612	2726	N/A	N/A	N/A	N/A
Follow-up	16 Aug	7763	3215	7752	2738	11.19 (1,113)	.001 c	1.18 (1,113)	.28
	16 Sep	7857	2893	8090	2559	7.17 (1,113)	.009 c	0.30 (1,113)	.58
	16 Oct	8271	3310	8379	2532	0.25 (1,113)	.62	0.84 (1,113)	.36
	16 Nov	8700	3394	8199	2573	0.04 (1,113)	.84	4.97 (1,113)	.03 c
	16 Dec	8603	3448	8076	2394	0.11 (1,113)	.74	5.20 (1,113)	.02 c
	17 Jan	8551	3550	8030	2504	0.23 (1,113)	.63	3.67 (1,113)	.06
	17 Feb	8799	3436	8097	2494	0.04 (1,113)	.84	7.73 (1,113)	.006 c
	17 Mar a	8434	3015	8145	2390	0.33 (1,113)	.57	2.88 (1,113)	.09

[a]Significant violation of homogeneity of variance assumption between groups (Levene's test: $P < .05$)

[b]Significantly deviated from normality (Shapiro–Wilk test: $P < .05$), [c]$P < .05$

Table A2. Results of a 2-way repeated-measures analysis of variance (Female; N=115)

Time point		Players (n=23)		Nonplayers (n=92)		Time effect		Interaction term (time×player)	
		Mean	SD	Mean	SD	*F* test (*df*)	*P* value	*F* test (*df*)	*P* value
Baseline	16 Jun	7082	2236	7194	2437	N/A	N/A	N/A	N/A
Follow-up	16 Aug a	6651	2465	6373 b	2184	11.86 (1,113)	<.001	1.15 (1,113)	.29
	16 Sep	6804	2077	6677	2192	8.86 (1,113)	.004 c	0.81 (1,113)	.37
	16 Oct	7306	2019	7046	2278	0.07 (1,113)	.78	1.80 (1,113)	.18
	16 Nov	7149	2404	7109	2311	0.00 (1,113)	.95	0.22 (1,113)	.64
	16 Dec a	7417	2264	6778	2309	0.08 (1,113)	.78	6.74 (1,113)	.01 c
	17 Jan	6675	1992	6656	2338	9.21 (1,113)	.003 c	0.18 (1,113)	.67
	17 Feb	6941	2423	6803	2339	2.30 (1,113)	.13	0.51 (1,113)	.48
	17 Mar	6659	2441	6651	2378	9.55 (1,113)	.003 c	0.15 (1,113)	.70

[a]Significant violation of homogeneity of variance assumption between groups (Levene's test: $P < .05$)

[b]Significantly deviated from normality (Shapiro–Wilk test: $P < .05$), [c]$P < .05$

Table A3. Results of a 2-way repeated-measures analysis of variance (Aged <55; N=100)

Time point		Players (n=20)		Nonplayers (n=80)		Time effect		Interaction term (time×player)	
		Mean	SD	Mean	SD	*F* test (*df*)	*P* value	*F* test (*df*)	*P* value
Baseline	16 Jun a	7337	2700	8039	2403	N/A	N/A	N/A	N/A
Follow-up	16 Aug	6967	2975	7270	2248	7.25 (1,98)	.008 c	0.89 (1,98)	.35
	16 Sep	7149	2822	7603	2287	3.96 (1,98)	.05 c	0.63 (1,98)	.43
	16 Oct	6882	2485	7749	2329	5.62 (1,98)	.02 c	0.27 (1,98)	.60
	16 Nov a	7256	2975	7842	2542	0.48 (1,98)	.49	0.08 (1,98)	.77
	16 Dec	7160	2288	7516	2231	4.26 (1,98)	.04 c	1.04 (1,98)	.31
	17 Jan a	6873	2372	7384	2268	8.98 (1,98)	.003 c	0.26 (1,98)	.61
	17 Feb a	7038	2388	7603	2345	4.08 (1,98)	.05 c	0.14 (1,98)	.71
	17 Mar a	6891	2363	7560	2303	6.04 (1,98)	.02 c	0.01 (1,98)	.93

[a]Significant violation of homogeneity of variance assumption between groups (Levene's test: $P < .05$)

[b]Significantly deviated from normality (Shapiro–Wilk test: $P < .05$), [c]$P < .05$

Table A4. Results of a 2-way repeated-measures analysis of variance (Aged 55-64; N=80)

Time point		Players (n=16)		Nonplayers (n=64)		Time effect		Interaction term (time×player)	
		Mean	SD	Mean	SD	*F* test (*df*)	*P* value	*F* test (*df*)	*P* value
Baseline	16 Jun	8553	2476	8234	2995	N/A	N/A	N/A	N/A
Follow-up	16 Aug a	8628	2277	7288 b	2826	3.66 (1,78)	.06	5.02 (1,78)	.03 c
	16 Sep	8297	2064	7592	2607	5.60 (1,78)	.02 c	1.03 (1,78)	.31
	16 Oct a	9437	2500	8029	2571	2.99 (1,78)	.09	7.68 (1,78)	.007 c
	16 Nov a	9518	2493	7916	2494	2.13 (1,78)	.15	8.37 (1,78)	.005 c
	16 Dec a	9654	2353	7763	2609	1.88 (1,78)	.17	11.73 (1,78)	<.001
	17 Jan	9163	2586	7587	2661	0.00 (1,78)	.94	5.46 (1,78)	.02 c
	17 Feb a	9517	2750	7807	2582	1.09 (1,78)	.30	7.36 (1,78)	.008 c
	17 Mar	8986	2534	7531	2643	0.34 (1,78)	.56	5.96 (1,78)	.02 c

[a]Significant violation of homogeneity of variance assumption between groups (Levene's test: $P < .05$)
[b]Significantly deviated from normality (Shapiro–Wilk test: $P < .05$), [c]$P < .05$

Table A5. Results of a 2-way repeated-measures analysis of variance (Aged 65+; N=50)

Time point		Players (n=10)		Nonplayers (n=40)		Time effect		Interaction term (time×player)	
		Mean	SD	Mean	SD	*F* test (*df*)	*P* value	*F* test (*df*)	*P* value
Baseline	16 Jun	6793	3139	7102	2551	N/A	N/A	N/A	N/A
Follow-up	16 Aug	5414	2664	6287	2634	21.38 (1,48)	<.001	1.41 (1,48)	.24
	16 Sep a	6147	2283	6610	2546	7.61 (1,48)	.008 c	0.14 (1,48)	.71
	16 Oct	6963	2658	7133	2645	0.25 (1,48)	.62	0.12 (1,48)	.73
	16 Nov	6709	3028	6857	2311	0.36 (1,48)	.55	0.09 (1,48)	.77
	16 Dec	7079	3966	6712	2449	0.03 (1,48)	.85	1.46 (1,48)	.23
	17 Jan	6613	3922	6871 b	2725	0.37 (1,48)	.55	0.01 (1,48)	.94
	17 Feb	6898	3947	6574	2510	0.62 (1,48)	.43	1.40 (1,48)	.24
	17 Mar	6555	3549	6860 b	2598	0.80 (1,48)	.38	0.00 (1,48)	.99

[a]Significant violation of homogeneity of variance assumption between groups (Levene's test: $P < .05$)
[b]Significantly deviated from normality (Shapiro–Wilk test: $P < .05$), [c]$P < .05$

Table A6. Results of a 2-way repeated-measures analysis of variance (Worker; N=147)

Time point		Players (n=33)		Nonplayers (n=114)		Time effect		Interaction term (time×player)	
		Mean	SD	Mean	SD	*F* test (*df*)	*P* value	*F* test (*df*)	*P* value
Baseline	16 Jun	7821	2801	8222	2705	N/A	N/A	N/A	N/A
Follow-up	16 Aug a	7653	2785	7529 b	2539	8.28 (1,145)	.005 c	3.07 (1,145)	.08
	16 Sep	7661	2671	7767	2419	6.13 (1,145)	.01 c	1.39 (1,145)	.24
	16 Oct	7855	2926	7925	2408	1.09 (1,145)	.30	1.71 (1,145)	.19
	16 Nov	8265	3129	7951	2543	0.31 (1,145)	.58	5.32 (1,145)	.02 c
	16 Dec	8021	2895	7756	2359	0.86 (1,145)	.35	5.38 (1,145)	.02 c
	17 Jan	7746	2988	7575	2349	4.93 (1,145)	.03 c	3.08 (1,145)	.08
	17 Feb	7991	3004	7829	2437	0.53 (1,145)	.47	3.36 (1,145)	.07
	17 Mar	7840	2805	7783	2383	2.11 (1,145)	.15	2.50 (1,145)	.12

[a]Significant violation of homogeneity of variance assumption between groups (Levene's test: $P < .05$)
[b]Significantly deviated from normality (Shapiro–Wilk test: $P < .05$), [c]$P < .05$

Table A7. Results of a 2-way repeated-measures analysis of variance (Non-worker; N=83)

Time point		Players (n=13)		Nonplayers (n=70)		Time effect		Interaction term (time×player)	
		Mean	SD	Mean	SD	*F* test (*df*)	*P* value	*F* test (*df*)	*P* value
Baseline	16 Jun	7186	2688	7385	2560	N/A	N/A	N/A	N/A
Follow-up	16 Aug	6076	2944	6304	2438	17.72 (1,81)	<.001	0.00 (1,81)	.96
	16 Sep a	6493	2057	6758	2466	11.59 (1,81)	.001 c	0.03 (1,81)	.87
	16 Oct	7619	2363	7367	2606	1.04 (1,81)	.31	1.23 (1,81)	.27
	16 Nov	7059	2604	7171	2367	0.48 (1,81)	.49	0.03 (1,81)	.86
	16 Dec	7983	3191	6891 b	2476	0.38 (1,81)	.54	6.78 (1,81)	.01 c
	17 Jan	7276	3127	6966 b	2733	0.31 (1,81)	.58	0.76 (1,81)	.39
	17 Feb	7563	3394	6832	2486	0.11 (1,81)	.74	3.13 (1,81)	.08
	17 Mar	6803	2965	6770 b	2556	3.56 (1,81)	.06	0.19 (1,81)	.66

[a]Significant violation of homogeneity of variance assumption between groups (Levene's test: $P < .05$)
[b]Significantly deviated from normality (Shapiro–Wilk test: $P < .05$), [c]$P < .05$

Table A8. Results of a 2-way repeated-measures analysis of variance (PA level: active; N=85)

Time point		Players (n=17)		Nonplayers (n=68)		Time effect		Interaction term (time×player)	
		Mean	SD	Mean	SD	*F* test (*df*)	*P* value	*F* test (*df*)	*P* value
Baseline	16 Jun	7001	3250	8218	2470	N/A	N/A	N/A	N/A
Follow-up	16 Aug	6916	3093	7186 b	2302	7.29 (1,83)	.008 c	5.24 (1,83)	.02 c
	16 Sep	6944	2689	7620	2214	3.85 (1,83)	.05	2.62 (1,83)	.11
	16 Oct	7450	2758	7865	2429	0.08 (1,83)	.77	5.87 (1,83)	.02 c
	16 Nov a	7420	2689	8023	2499	0.35 (1,83)	.55	2.66 (1,83)	.11
	16 Dec	7589	2785	7776	2382	0.13 (1,83)	.72	6.66 (1,83)	.01 c
	17 Jan	7042	2943	7772	2600	0.76 (1,83)	.39	1.10 (1,83)	.30
	17 Feb a	7497	3324	7895	2472	0.15 (1,83)	.70	3.34 (1,83)	.07
	17 Mar	7496	2877	7709 b	2566	0.00 (1,83)	.97	5.26 (1,83)	.02 c

[a]Significant violation of homogeneity of variance assumption between groups (Levene's test: $P < .05$)
[b]Significantly deviated from normality (Shapiro–Wilk test: $P < .05$), [c]$P < .05$

Table A9. Results of a 2-way repeated-measures analysis of variance (PA level: non-active; N=145)

Time point		Players (n=29)		Nonplayers (n=116)		Time effect		Interaction term (time×player)	
		Mean	SD	Mean	SD	*F* test (*df*)	*P* value	*F* test (*df*)	*P* value
Baseline	16 Jun	8017	2401	7719	2782	N/A	N/A	N/A	N/A
Follow-up	16 Aug	7377	2803	6990	2714	16.02 (1,143)	<.001	0.07 (1,143)	.80
	16 Sep	7557	2479	7245	2622	12.16 (1,143)	<.001	0.00 (1,143)	.96
	16 Oct	7987	2781	7623	2536	0.20 (1,143)	.65	0.06 (1,143)	.81
	16 Nov	8220	3194	7437	2485	0.05 (1,143)	.82	1.87 (1,143)	.17
	16 Dec	8257	3057	7223	2450	0.63 (1,143)	.43	5.20 (1,143)	.02 c
	17 Jan	7948	3034	7092	2435	3.54 (1,143)	.06	2.27 (1,143)	.13
	17 Feb	8089	2976	7190	2484	1.91 (1,143)	.17	3.28 (1,143)	.07
	17 Mar	7577	2896	7216	2442	8.81 (1,143)	.004 c	0.04 (1,143)	.84

[a]Significant violation of homogeneity of variance assumption between groups (Levene's test: $P < .05$)
[b]Significantly deviated from normality (Shapiro–Wilk test: $P < .05$), [c]$P < .05$

Table A10. Results of a 2-way repeated-measures analysis of variance (Health status: good; N=82)

Time point		Players (n=17)		Nonplayers (n=65)		Time effect		Interaction term (time×player)	
		Mean	SD	Mean	SD	*F* test (*df*)	*P* value	*F* test (*df*)	*P* value
Baseline	16 Jun a	8412	2309	8553 b	2376	N/A	N/A	N/A	N/A
Follow-up	16 Aug a	7829	2571	7749 b	2418	10.18 (1,80)	.002 c	0.26 (1,80)	.61
	16 Sep	7984	2078	7935	2203	6.86 (1,80)	.01 c	0.22 (1,80)	.64
	16 Oct	8626	2358	8326	2215	0.00 (1,80)	.97	1.51 (1,80)	.22
	16 Nov a	9162	2816	8174	2220	0.61 (1,80)	.44	5.60 (1,80)	.02 c
	16 Dec a	8973	2835	7908	2197	0.03 (1,80)	.86	6.66 (1,80)	.01 c
	17 Jan a	8613	3303	7930	2260	0.72 (1,80)	.40	2.76 (1,80)	.10
	17 Feb	8721	3074	8046 b	2311	0.21 (1,80)	.64	3.65 (1,80)	.06
	17 Mar	8357	2670	8086	2289	1.34 (1,80)	.25	0.84 (1,80)	.36

[a]Significant violation of homogeneity of variance assumption between groups (Levene's test: $P < .05$)
[b]Significantly deviated from normality (Shapiro–Wilk test: $P < .05$), [c]$P < .05$

Table A11. Results of a 2-way repeated-measures analysis of variance (Health status: others; N=148)

Time point		Players (n=29)		Nonplayers (n=119)		Time effect		Interaction term (time×player)	
		Mean	SD	Mean	SD	*F* test (*df*)	*P* value	*F* test (*df*)	*P* value
Baseline	16 Jun a	7190	2928	7549	2770	N/A	N/A	N/A	N/A
Follow-up	16 Aug	6843	3042	6688 b	2575	12.95 (1,146)	<.001	2.33 (1,146)	.13
	16 Sep	6948	2746	7082	2577	8.86 (1,146)	.003 c	0.89 (1,146)	.35
	16 Oct	7298	2888	7377	2580	0.06 (1,146)	.81	1.06 (1,146)	.31
	16 Nov	7198	2928	7370	2605	0.31 (1,146)	.58	0.37 (1,146)	.54
	16 Dec	7446	2909	7164	2524	0.20 (1,146)	.66	4.84 (1,146)	.03 c
	17 Jan	7027	2696	7023	2593	3.74 (1,146)	.05	1.03 (1,146)	.31
	17 Feb	7371	3035	7124	2543	0.51 (1,146)	.48	3.13 (1,146)	.08
	17 Mar	7072	2901	7022 b	2529	4.14 (1,146)	.04 c	1.65 (1,146)	.20

[a]Significant violation of homogeneity of variance assumption between groups (Levene's test: $P < .05$)
[b]Significantly deviated from normality (Shapiro–Wilk test: $P < .05$), [c]$P < .05$
